# Synthesis, crystal structure and thermal reactivity of di­aqua­dithio­cyanato­bis­(pyridine-4-carbo­nitrile)cobalt(II)

**DOI:** 10.1107/S2056989026007371

**Published:** 2026-07-23

**Authors:** Christian Näther

**Affiliations:** a Institut für Anorganische Chemie, Universität Kiel, Max-Eyth.-Str. 2, 24118 Kiel, Germany; University of Buenos Aires, Argentina

**Keywords:** synthesis, crystal structure, cobalt thio­cyanate, aqua complex, 4-cyano­pyridine, hydrogen bonding, thermal properties

## Abstract

The title compound, [Co(NCS)_2_(C_6_H_4_N_2_)_2_(H_2_O)_2_], is isotypic to its Ni analog and to one of the two modifications of Mn(NCS)_2_(4-cyano­pyridine)(H_2_O)_2_ reported recently [Näther (2026 ▸). *Acta Cryst.* E**82**, 441–445 and Wellm *et al.* (2020 ▸). *Cryst. Growth Des.***20**, 3374–3385]. Its asymmetric unit is built up of one cobalt cation that occupies a center of inversion, one thio­cyanate anion, one neutral 4-cyano­pyridine coligand and one water mol­ecule that are located in general positions. The metal cations are sixfold coordinated by two terminally N-bonding thio­cyanate anions, two water mol­ecules and two 4-cyano­pyridine coligands within a slightly distorted octa­hedral geometry. The discrete complexes are linked by inter­molecular O—H⋯S hydrogen bonding into layers that condense into a three-dimensional network *via* weak C—H⋯N inter­actions. Powder X-ray diffraction (PXRD) proves that a pure sample has been obtained. Thermogravimetric measurements reveal that the title compound decomposes in different steps, in which compounds with the composition Co(NCS)_2_(4-cyano­pyridine)_2_ and Co(NCS)_2_(4-cyano­pyridine) are formed as inter­mediates, which are not isotypic to the corresponding Mn compounds already reported in the literature [Wellm *et al.* (2020 ▸). *Cryst. Growth Des.***20**, 3374–3385].

## Abstract 2

In the crystal structure of the title compound, the cobalt cations are octa­hedrally coordinated by two terminal N-bonding thio­cyanate anions, two 4-cyano­pyridine ligands and two water mol­ecules into discrete complexes that are linked by inter­molecular O—H⋯S hydrogen bonding into layers that condense into a three-dimensional hydrogen bonded network by weak inter­molecular C—H⋯N inter­actions.

## 1. Chemical context

The synthesis of new coordination compounds with desired physical properties is still a major goal in coordination chemistry (Ferrando-Soria *et al.*, 2017 ▸; Yue & Gao, 2019 ▸). In most cases, such compounds are prepared in solution but there are alternatives such as, for example, mol­ecular milling (Braga *et al.*, 2005 ▸, 2006 ▸; James *et al.*, 2012 ▸; Do & Friščić, 2017 ▸; Stolar *et al.*, 2017 ▸) or reactions in melts (Müller-Buschbaum, 2005 ▸; Höller & Müller-Buschbaum, 2008 ▸; Zurawski *et al.*, 2012 ▸), which represent typical solid-state synthesis methods. Many years ago, we established a further very simple method that can be used for the synthesis of coordination compounds with condensed coordination networks. In the beginning, this method was used to prepare transition-metal halide and pseudohalide coordination compounds (Näther *et al.*, 2002 ▸) but it was later expanded to coordination compounds based on transition-metal thio- and seleno­cyanates (Näther & Greve, 2003 ▸; Wriedt & Näther, 2010 ▸; Wöhlert *et al.*, 2012 ▸). Following this route, simple precursor compounds such as discrete complexes with terminally thio- or seleno­cyanate anions are heated, which frequently leads to a stepwise removal of the neutral coligands and the formation of coligand-deficient inter­mediate phases in which the metal cations are linked by bridging anionic ligands. These inter­mediate compounds are of inter­est because they can show inter­esting magnetic properties that are mediated by the bridging anionic ligands (Wöhlert *et al.*, 2013 ▸; Neumann *et al.*, 2018 ▸). In this context, compounds based on Co(NCS)_2_ or Co(NCSe)_2_ are of special inter­est, because they can show 1D and 3D magnetic ordering (Lescouëzec *et al.*, 2005 ▸; Mautner *et al.*, 2018 ▸; Rams *et al.*, 2020 ▸).

In the course of our work, we investigated the influence of the coligand, which mostly consists of pyridine derivatives, because in this case the thermal treatment of the precursor leads to compounds with 1D or 2D thio- or seleno­cyanate networks. Within this project we became inter­ested in 4-cyano­pyridine as coligand, which can in principle also act as bridging ligand and for which some compounds were already reported (see *Database survey*). With Mn(NCS)_2_ we obtained a large number of compounds with different ratios between Mn(NCS)_2_ and 4-cyano­pyridine (Wellm *et al.*, 2020 ▸), including the discrete complex Mn(NCS)_2_(4-cyano­pyridine)_4_ (refcode OJEDOZ; Wellm *et al.*, 2020 ▸) as well as two different modifications of Mn(NCS)_2_(4-cyano­pyridine)_2_(H_2_O)_2_ (OJEFAN and OJEFAN01; Wellm *et al.*, 2020 ▸), one of which is isotypic to the title compound and to Ni(NCS)_2_(4-cyano­pyridine)_2_(H_2_O)_2_ reported recently (Näther, 2026 ▸). Two further aqua complexes of same composition but with additional 4-cyano­pyridine as solvate ligand were also obtained (OJEFER and OJEFUH; Wellm *et al.*, 2020 ▸). If the aqua complex is heated, a transformation is observed into Mn(NCS)_2_(4-cyano­pyridine)_2_ (OJEFIV; Wellm *et al.*, 2020 ▸), which transforms into Mn(NCS)_2_(4-cyano­pyridine) (OJEDUF and OJEDUF01; Wellm *et al.*, 2020 ▸) upon further heating. For the latter compound, two different isomers were obtained.

To investigate whether a corresponding aqua complex with Co(NCS)_2_ is available and if it will show a similar reactivity to that with Mn(NCS)_2_, we reacted Co(NCS)_2_ with 4-cyano­pyridine using similar conditions as for the synthesis of Mn(NCS)_2_(4-cyano­pyridine)_2_(H_2_O)_2_. The crystals obtained by this procedure were characterized by single-crystal X-ray diffraction.
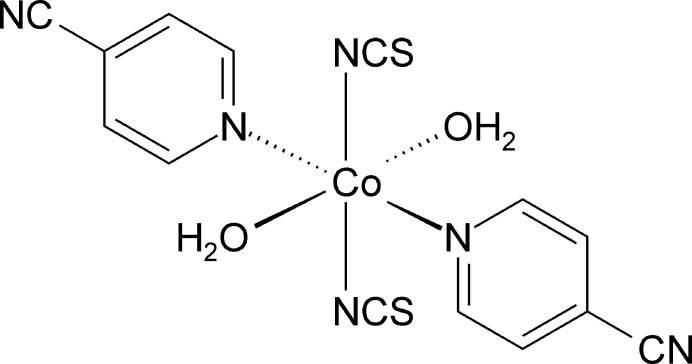


## 2. Structural commentary

The title compound Co(NCS)_2_(C_6_H_4_N_2_)_2_(H_2_O)_2_ (C_6_H_4_N_2_ = 4-cyano­pyridine) is isotypic to Ni(NCS)_2_(4-cyano­pyridine)_2_(H_2_O)_2_ (Näther, 2026 ▸) reported recently and to one of the two modifications of Mn(NCS)_2_(4-cyano­pyridine)_2_(H_2_O)_2_ (OJEFAN; Wellm *et al.*, 2020 ▸).

**Figure 1:**
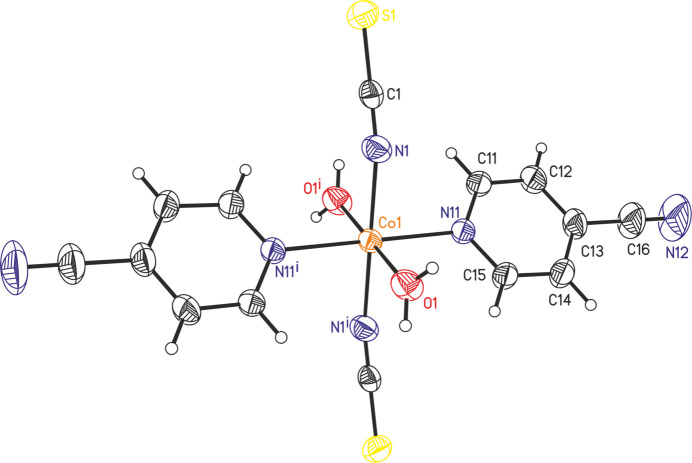
Crystal structure of the title compound with labeling and displacement ellipsoids drawn at the 50% probability level. Symmetry code: (i) −*x* + 1, −*y* + 1, −*z*.

The asymmetric unit consists of one Co cation that is located on a center of inversion, as well as one thio­cyanate anion, one 4-cyano­pyridine ligand and one water mol­ecule that are located in general positions. The metal cations are sixfold coordinated by two terminally N-bonding thio­cyanate anions, two water mol­ecules and two 4-cyano­pyridine ligands that coordinate with the pyridine N atom to the metal center (Fig. 1 ▸). This arrangement leads to the formation of discrete complexes, which show a slightly distorted octa­hedral coordination (see supporting information).

## 3. Supra­molecular features

**Figure 2:**
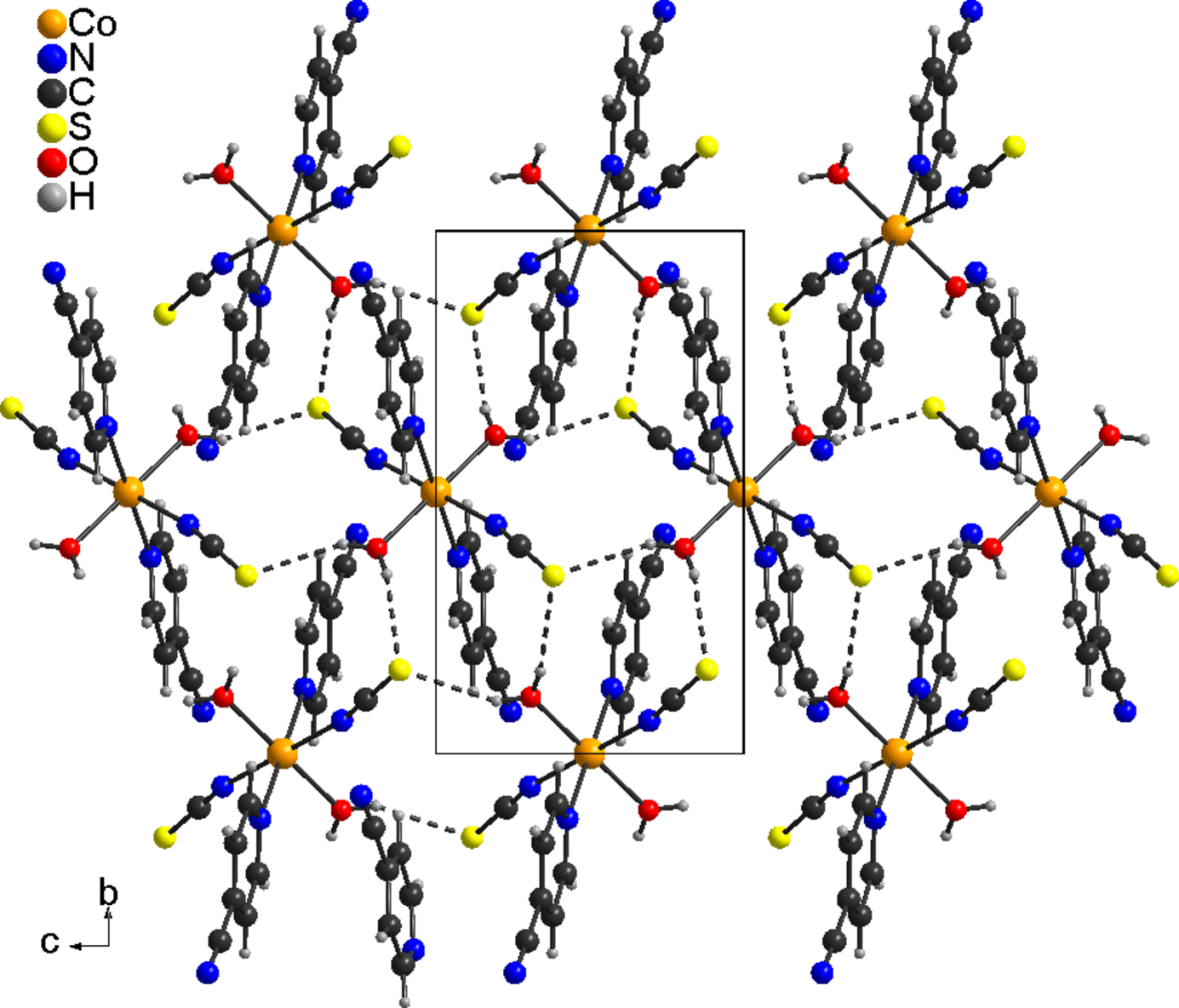
Crystal structure of the title compound in a view along the crystallographic *a*-axis direction with inter­molecular O—H⋯S hydrogen bonding shown as dashed lines.

In the crystal structure of the title compound, the discrete complexes are connected *via* inter­molecular O—H⋯S hydrogen bonding between the thio­cyanate S atom and the H atoms of the water mol­ecules. Each water mol­ecule is involved in two hydrogen bonds to S atoms of neighboring complexes and each S atom acts as acceptor for two hydrogen bonds to two different water mol­ecules (Fig. 2 ▸). This arrangement leads to the formation of ten-membered hydrogen-bonded rings involving three symmetry-related complexes. These rings condense into layers that are parallel to the *bc* plane (Fig. 2 ▸). The O–H⋯S angles are close to linearity, indicating relatively strong inter­actions (Table 1 ▸).

**Figure 3:**
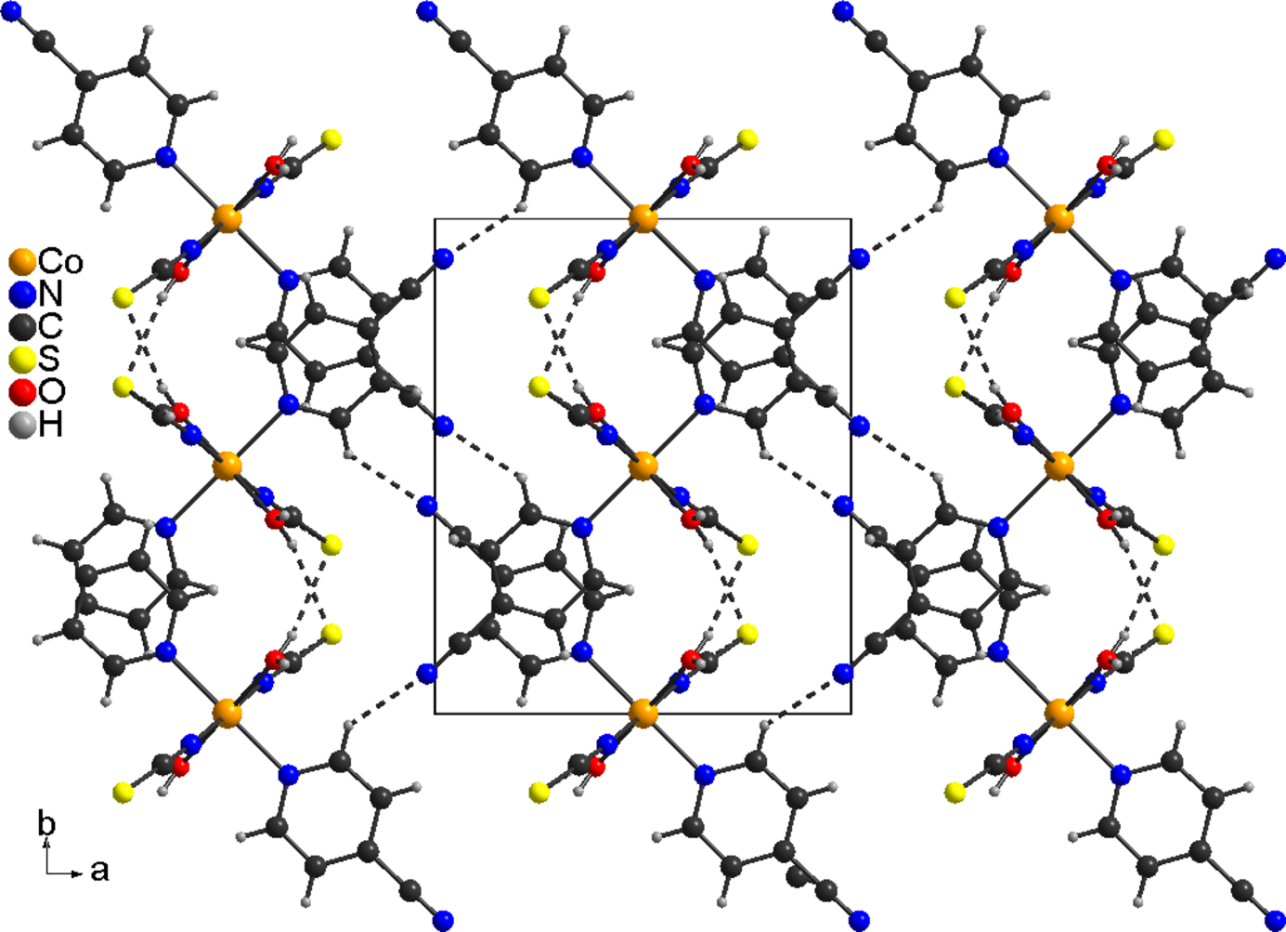
Crystal structure of the title compound in a view along the crystallographic *c*-axis direction with inter­molecular O—H⋯S and C—H⋯N hydrogen bonding shown as dashed lines.

The layers are linked by pairs of C—H⋯N inter­actions between the pyridine N atom of one complex and the cyano N atom of a neighboring complex, forming hydrogen-bonded rings that are located around centers of inversion (Fig. 3 ▸). From this inter­action, a three-dimensional network is formed (Fig. 3 ▸). The C—H⋯N angle is far from linear, indicating a weak inter­action (Table 1 ▸).

## 4. Additional characterization

**Figure 4:**
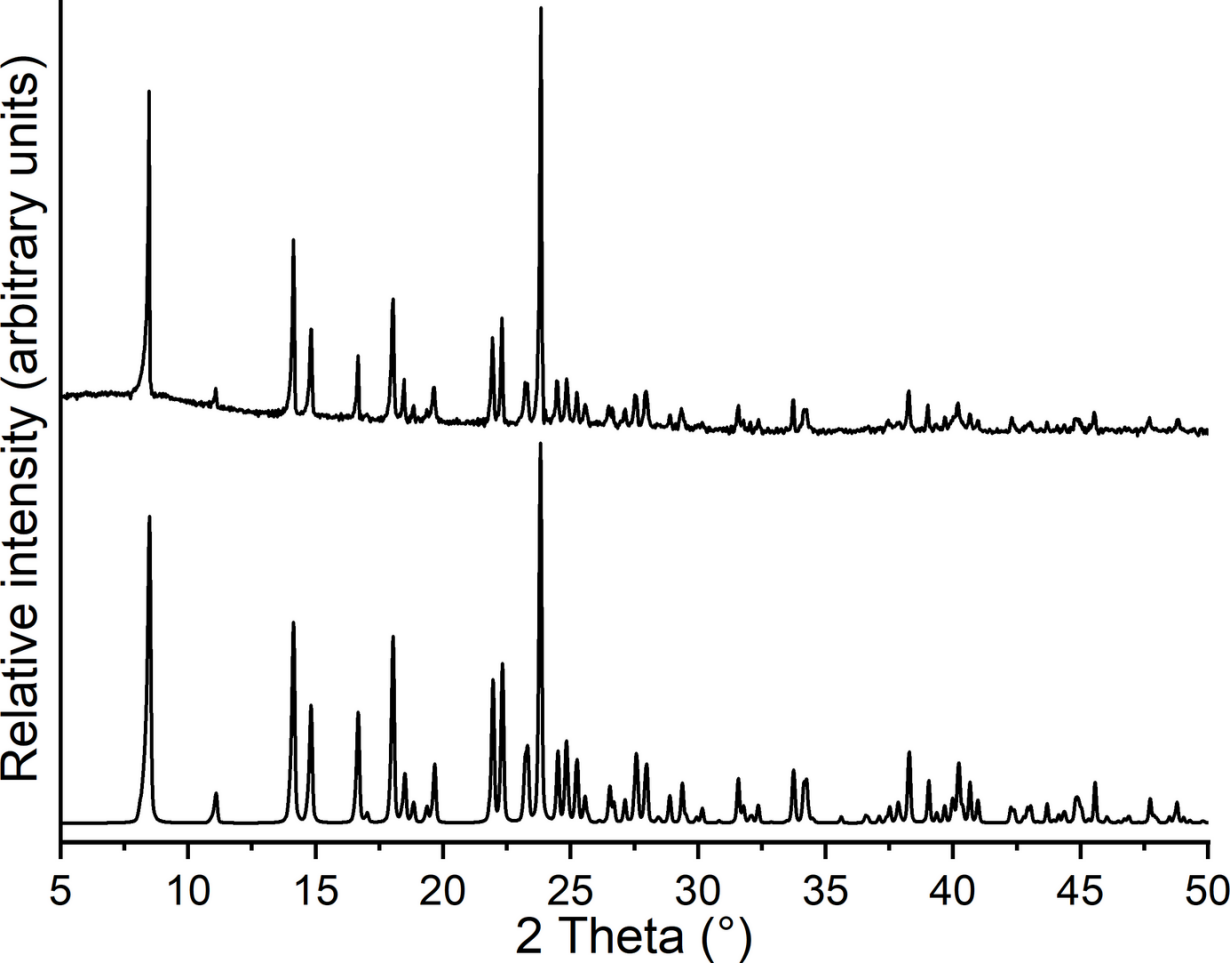
Experimental (top) and calculated (bottom) PXRD patterns for the title compound.

The experimental powder pattern of crystals of the title compound was compared with that calculated from single-crystal data, proving that a pure crystalline phase had been obtained (Fig. 4 ▸). The CN stretching vibration of the thio­cyanate anion is observed at 2094 cm^−1^ in the IR spectrum, in agreement with terminal coordinated anionic ligands and the presence of water is obvious from the strong band at 3343 cm^−1^ (Fig. S1).

**Figure 5:**
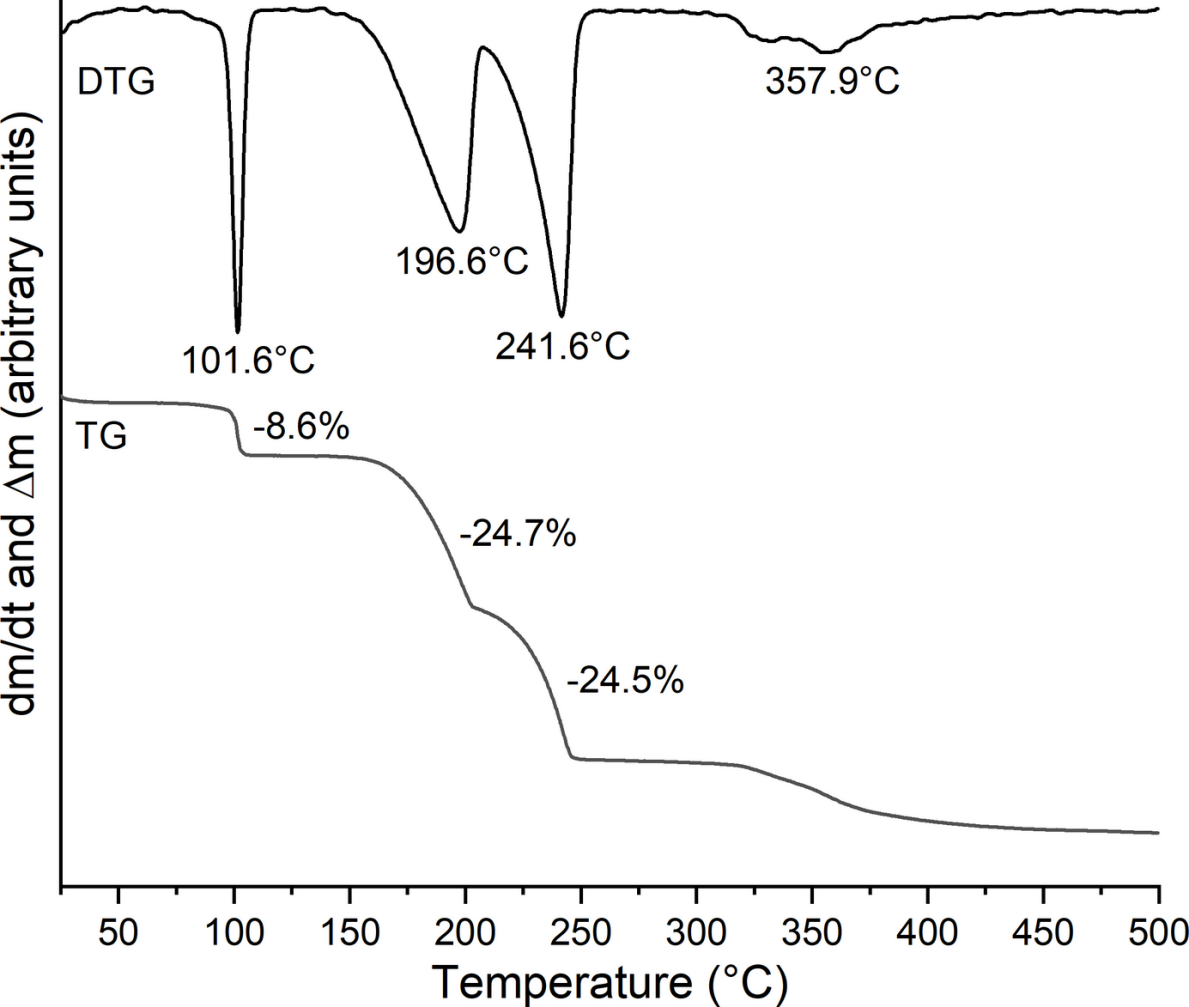
DTG and TG curves for the title compound. The mass loss is given together with the peak temperature (in °C).

Investigations using thermogravimetry (DTA-TG) reveal that the title compound decomposes in three different reasonably resolved steps (Fig. 5 ▸). The first mass loss of 8.6% is in perfect agreement with that calculated for the loss of the two water mol­ecules (8.6%). Upon further heating, two similar steps of 24.7 and 24.5% are observed, which should each correspond to the loss of one 4-cyano­pyridine ligand in each step (Δ*m*_calc._: 24.8%). Therefore, one can assume that after the first mass loss a compound with the composition Co(NCS)_2_(4-cyano­pyridine)_2_ is formed, which decomposes into Co(NCS)_2_)(4-cyano­pyridine) upon further heating. After the third mass loss Co(NCS)_2_ is formed. It is noted, that similar results are reported for the thermal reactivity of Mn(NCS)_2_(4-cyano­pyridine)_2_(H_2_O)_2_ (Wellm *et al.*, 2020 ▸).

To further characterize the inter­mediate compounds obtained by thermogravimetry, the residues isolated after the first and second mass loss were investigated by powder X-ray diffraction. Comparison of the experimental pattern of the residue obtained after the first step with that calculated for Mn(NCS)_2_(4-cyano­pyridine)_2_ retrieved from the literature (Wellm *et al.*, 2020 ▸) indicates that this crystalline phase is present and that additional reflections are observed, indicating for a further crystalline phase (Fig. S2). However, the overall quality of the pattern is relatively low, indicating that a poorly crystalline powder was obtained. If the powder pattern of the residue obtained after the second mass loss is compared with that calculated for both isomers of Mn(NCS)_2_(4-cyano­pyridine), it is obvious that a completely different and unknown crystalline phase has formed (Fig. S3).

## 5. Synthesis and crystallization

**General:**

Co(NCS)_2_ and 4-cyano­pyridine were purchased from Sigma-Aldrich.

**Synthesis:**

2 mmol (354.2 mg) Co(NCS)_2_ and 4 mmol (416.4 mg) 4-cyano­pyridine were reacted in 3 mL of water. Within 2 d, crystals suitable for crystal structure analysis were obtained.

**Experimental details**

Powder X-ray diffraction measurements were performed using a Stoe STADI P transmission powder diffractometer with Cu *K*α_1_ radiation (λ= 1.540598 Å), equipped with a Johann-type Ge(111) monochromator and a MYTHEN 1K detector from Dectris.

Thermogravimetry measurements were performed in a dynamic nitro­gen atmosphere in Al_2_O_3_ crucibles with a heating rate of 4 °C min^−1^ using a STA-PT 1000 thermobalance from Linseis.

IR spectroscopy was done using an ATI Mattson Genesis Series FTIR Spectrometer, control software: *WINFIRST*, from ATI Mattson in ATR mode.

## 6. Database survey

A search in the Cambridge Structural Database (CSD Version 5.43, last update January 2026; Groom *et al.*, 2016 ▸) using CONQUEST (Bruno *et al.*, 2002 ▸) revealed that some compounds with 4-cyano­pyridine and transition metal cations are already published. These include discrete complexes with the composition Ni(NCS)_2_(4-cyano­pyridine)_4_ (UBUBOL; Clegg & Harrington, 2016 ▸) and Mn(NCS)_2_(4-cyano­pyridine)_4_ (OJEDOZ; Wellm *et al.*, 2020 ▸) that are not isotypic as well as aqua complexes with the composition Ni(NCS)_2_(4-cyano­pyridine)_2_(H_2_O)_2_ (YANHAB; Näther, 2026 ▸) and two different modifications of Mn(NCS)_2_(4-cyano­pyridine)_2_(H_2_O)_2_ (OJEFAN and OJEFAN01; Wellm *et al.*, 2020 ▸) one of which is isotypic to the nickel and the title compounds. Discrete complexes with additional 4-cyano­pyridine as solvate are also known, including the isotypic compounds Fe(NCS)_2_(4-cyano­pyridine)_2_(H_2_O)_2_·(4-cyano­pyridine)_2_ (KAPQEA; Jochim *et al.*, 2017 ▸) and Mn(NCS)_2_(4-cyano­pyridine)_2_(H_2_O)_2_·(4-cyano­pyridine)_2_ (OJEFUH; Wellm *et al.*, 2020 ▸) as well as Mn(NCS)_2_(4-cyano­pyridine)_2_(H_2_O)_2_·(4-cyano­pyridine)_4_ (OJEFER; Wellm *et al.*, 2020 ▸). Compounds with 4-cyano­pyridine and bridging thio­cyanate anions are also reported. They include Cd(NCS)_2_(4-cyano­pyridine)_2_ in which the metal cations are linked into chains (WUCLUB; Chen *et al.*, 2002 ▸) and Cd(NCS)_2_(4-cyano­pyridine) (WUCMAI; Chen *et al.*, 2002 ▸) in which the 4-cyano­pyridine ligand acts as a bridging ligand. Finally, Cu(NCS)_2_(4-cyano­pyridine)_2_ is also found, which exhibits a structure similar to that of the corresponding Mn compound (ABOVOF; Handy *et al.*, 2017 ▸).

## 7. Refinement

Crystal data, data collection and structure refinement details are summarized in Table 2 ▸. The C—H hydrogen atoms were positioned with idealized geometry and were refined with *U*_iso_(H) = 1.2*U*_eq_(C) using a riding model. The O—H hydrogen atoms were located in difference maps and were refined isotropic with *U*_iso_(H) = 1.5*U*_eq_(O) using restraints.

## Supplementary Material

Crystal structure: contains datablock(s) I. DOI: 10.1107/S2056989026007371/vu2019sup1.cif

Structure factors: contains datablock(s) I. DOI: 10.1107/S2056989026007371/vu2019Isup2.hkl

Figure S1. IR spectrum of the title compound. Given is the O-H stretching vibration of the water molecules and the CN stretching vibration of the thiocyanate anions. DOI: 10.1107/S2056989026007371/vu2019sup3.png

Figure S2. X-ray powder pattern of the residue obtained after the first mass loss (top) and calculated powder pattern for Mn(NCS)2(4-cyanopyridine)2 (bottom) using data retrieved from literature (Wellm et al., 2020). DOI: 10.1107/S2056989026007371/vu2019sup4.png

Figure S3. X-ray powder pattern of the residue obtained after the second mass loss (top) and calculated powder pattern for both isomers of Mn(NCS)2(4-cyanopyridine) using data retrieved from literature (Wellm et al, 2020). DOI: 10.1107/S2056989026007371/vu2019sup5.png

CCDC reference: 2574033

Additional supporting information:  crystallographic information; 3D view; checkCIF report

## Figures and Tables

**Table 1:** Hydrogen-bond geometry (Å, °)

*D*—H⋯*A*	*D*—H	H⋯*A*	*D*⋯*A*	*D*—H⋯*A*
O1—H1*A*⋯S1^i^	0.82 (2)	2.50 (2)	3.2272 (17)	150 (3)
O1—H1*B*⋯S1^ii^	0.81 (2)	2.53 (2)	3.3227 (16)	168 (3)
C15—H15⋯N12^iii^	0.93	2.45	3.144 (3)	132

Symmetry codes: (i) 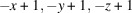; (ii) 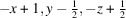; (iii) 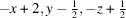.

**Table 2:** Experimental details

Crystal data	
Chemical formula	[Co(NCS)_2_(C_6_H_4_N_2_)_2_(H_2_O)_2_]
*M*_r_	419.35
Crystal system, space group	Monoclinic, *P*2_1_/*c*
Temperature (K)	298
*a*, *b*, *c* (Å)	10.7397 (5), 12.3497 (8), 7.4967 (4)
β (°)	104.596 (4)
*V* (Å^3^)	962.21 (9)
*Z*	2
Radiation type	Mo *K*α
μ (mm^−1^)	1.13
Crystal size (mm)	0.20 × 0.16 × 0.12

Data collection	
Diffractometer	Stoe IPDS2
Absorption correction	Numerical (*X-SHAPE* and *X-RED* 32; Stoe, 2008 ▸)
*T*_min_, *T*_max_	0.765, 0.910
No. of measured, independent and observed [*I* > 2σ(*I*)] reflections	15445, 2301, 2058
*R*_int_	0.031
(sin θ/λ)_max_ (Å^−1^)	0.660

Refinement	
*R*[*F*^2^ > 2σ(*F*^2^)], *wR*(*F*^2^), *S*	0.034, 0.085, 1.10
No. of reflections	2301
No. of parameters	121
No. of restraints	3
H-atom treatment	H atoms treated by a mixture of independent and constrained refinement
Δρ_max_, Δρ_min_ (e Å^−3^)	0.40, −0.29

Computer programs: *X-AREA* (Stoe, 2008 ▸), *SHELXT* (Sheldrick, 2015*a* ▸), *SHELXL* (Sheldrick, 2015*b* ▸), *DIAMOND* (Brandenburg, 1999 ▸), *XP* in *SHELXTL-PC* (Sheldrick, 2008 ▸) and *publCIF* (Westrip, 2010 ▸).

## supplementary crystallographic information

### Diaquadithiocyanatobis(pyridine-4-carbonitrile)cobalt(II) Crystal data

**Table:**

[Co(NCS)_2_(C_6_H_4_N_2_)_2_(H_2_O)_2_]	*F*(000) = 426
*M**_r_* = 419.35	*D*_x_ = 1.447 Mg m^−^^3^
Monoclinic, *P*2_1_/*c*	Mo *K*α radiation, λ = 0.71073 Å
*a* = 10.7397 (5) Å	Cell parameters from 8000 reflections
*b* = 12.3497 (8) Å	θ = 10.0–25.0°
*c* = 7.4967 (4) Å	µ = 1.13 mm^−^^1^
β = 104.596 (4)°	*T* = 298 K
*V* = 962.21 (9) Å^3^	Block, light blue
*Z* = 2	0.20 × 0.16 × 0.12 mm

### Diaquadithiocyanatobis(pyridine-4-carbonitrile)cobalt(II) Data collection

**Table:**

Stoe IPDS-2 diffractometer	2058 reflections with *I* > 2σ(*I*)
ω scans	*R*_int_ = 0.031
Absorption correction: numerical (X-Shape and X-Red 32; Stoe, 2008)	θ_max_ = 28.0°, θ_min_ = 2.6°
*T*_min_ = 0.765, *T*_max_ = 0.910	*h* = −12→14
15445 measured reflections	*k* = −16→16
2301 independent reflections	*l* = −9→9

### Diaquadithiocyanatobis(pyridine-4-carbonitrile)cobalt(II) Refinement

**Table:**

Refinement on *F*^2^	3 restraints
Least-squares matrix: full	Hydrogen site location: mixed
*R*[*F*^2^ > 2σ(*F*^2^)] = 0.034	H atoms treated by a mixture of independent and constrained refinement
*wR*(*F*^2^) = 0.085	*w* = 1/[σ^2^(*F*_o_^2^) + (0.0386*P*)^2^ + 0.2457*P*] where *P* = (*F*_o_^2^ + 2*F*_c_^2^)/3
*S* = 1.10	(Δ/σ)_max_ < 0.001
2301 reflections	Δρ_max_ = 0.40 e Å^−^^3^
121 parameters	Δρ_min_ = −0.29 e Å^−^^3^

### Diaquadithiocyanatobis(pyridine-4-carbonitrile)cobalt(II) Special details

**Table:**

Geometry. All esds (except the esd in the dihedral angle between two l.s. planes) are estimated using the full covariance matrix. The cell esds are taken into account individually in the estimation of esds in distances, angles and torsion angles; correlations between esds in cell parameters are only used when they are defined by crystal symmetry. An approximate (isotropic) treatment of cell esds is used for estimating esds involving l.s. planes.

### Diaquadithiocyanatobis(pyridine-4-carbonitrile)cobalt(II) Fractional atomic coordinates and isotropic or equivalent isotropic displacement parameters (Å^2^)

**Table:**

	*x*	*y*	*z*	*U*_iso_*/*U*_eq_	
Co1	0.500000	0.500000	0.000000	0.04781 (12)	
N1	0.41307 (18)	0.56286 (14)	0.1939 (3)	0.0650 (4)	
C1	0.34588 (18)	0.60302 (14)	0.2716 (3)	0.0517 (4)	
S1	0.24962 (5)	0.65987 (5)	0.38157 (8)	0.07134 (17)	
O1	0.61498 (16)	0.39204 (12)	0.1917 (2)	0.0700 (4)	
H1A	0.636 (3)	0.402 (2)	0.303 (2)	0.105*	
H1B	0.656 (3)	0.342 (2)	0.170 (4)	0.105*	
N11	0.64730 (14)	0.62443 (11)	0.0722 (2)	0.0541 (4)	
N12	1.0191 (3)	0.9194 (3)	0.2427 (7)	0.1738 (18)	
C11	0.6192 (2)	0.72943 (15)	0.0807 (3)	0.0632 (5)	
H11	0.533302	0.749637	0.059118	0.076*	
C12	0.7119 (2)	0.80896 (16)	0.1201 (4)	0.0730 (6)	
H12	0.689078	0.881551	0.121478	0.088*	
C13	0.8386 (2)	0.77856 (17)	0.1572 (4)	0.0722 (6)	
C14	0.8698 (2)	0.67080 (18)	0.1518 (4)	0.0733 (6)	
H14	0.955171	0.648522	0.177556	0.088*	
C15	0.77105 (18)	0.59709 (16)	0.1071 (3)	0.0643 (5)	
H15	0.791685	0.524276	0.101000	0.077*	
C16	0.9401 (3)	0.8582 (2)	0.2035 (6)	0.1074 (11)	

### Diaquadithiocyanatobis(pyridine-4-carbonitrile)cobalt(II) Atomic displacement parameters (Å^2^)

**Table:**

	*U*^11^	*U*^22^	*U*^33^	*U*^12^	*U*^13^	*U*^23^
Co1	0.04761 (19)	0.03533 (16)	0.0581 (2)	0.00129 (12)	0.00883 (14)	−0.00217 (13)
N1	0.0719 (11)	0.0523 (9)	0.0745 (11)	−0.0030 (8)	0.0254 (9)	−0.0088 (8)
C1	0.0549 (10)	0.0418 (8)	0.0557 (10)	−0.0082 (7)	0.0088 (8)	−0.0016 (7)
S1	0.0606 (3)	0.0821 (4)	0.0734 (4)	0.0073 (3)	0.0207 (3)	−0.0045 (3)
O1	0.0813 (10)	0.0519 (8)	0.0678 (9)	0.0139 (7)	0.0023 (8)	0.0019 (7)
N11	0.0483 (8)	0.0389 (7)	0.0707 (10)	0.0018 (6)	0.0070 (7)	−0.0040 (7)
N12	0.104 (2)	0.101 (2)	0.295 (5)	−0.0540 (19)	0.009 (3)	−0.033 (3)
C11	0.0528 (10)	0.0414 (9)	0.0910 (15)	0.0045 (8)	0.0097 (10)	−0.0084 (9)
C12	0.0699 (13)	0.0394 (9)	0.1041 (17)	−0.0022 (9)	0.0113 (12)	−0.0120 (10)
C13	0.0641 (12)	0.0533 (11)	0.0932 (16)	−0.0151 (9)	0.0087 (11)	−0.0103 (11)
C14	0.0476 (10)	0.0589 (11)	0.1054 (18)	−0.0009 (9)	0.0045 (11)	−0.0061 (12)
C15	0.0496 (10)	0.0420 (9)	0.0946 (15)	0.0039 (7)	0.0056 (10)	−0.0032 (9)
C16	0.0757 (17)	0.0678 (15)	0.167 (3)	−0.0211 (13)	0.0083 (19)	−0.0175 (18)

### Diaquadithiocyanatobis(pyridine-4-carbonitrile)cobalt(II) Geometric parameters (Å, º)

**Table:**

Co1—N1	2.0667 (18)	N11—C11	1.336 (2)
Co1—N1^i^	2.0668 (18)	N12—C16	1.118 (3)
Co1—O1	2.1150 (14)	C11—C12	1.376 (3)
Co1—O1^i^	2.1151 (14)	C11—H11	0.9300
Co1—N11^i^	2.1738 (15)	C12—C13	1.371 (3)
Co1—N11	2.1738 (15)	C12—H12	0.9300
N1—C1	1.148 (2)	C13—C14	1.375 (3)
C1—S1	1.634 (2)	C13—C16	1.445 (3)
O1—H1A	0.816 (17)	C14—C15	1.373 (3)
O1—H1B	0.805 (17)	C14—H14	0.9300
N11—C15	1.332 (2)	C15—H15	0.9300
			
N1—Co1—N1^i^	180.0	C15—N11—C11	117.55 (16)
N1—Co1—O1	92.79 (7)	C15—N11—Co1	119.82 (12)
N1^i^—Co1—O1	87.21 (7)	C11—N11—Co1	122.62 (13)
N1—Co1—O1^i^	87.21 (7)	N11—C11—C12	122.90 (19)
N1^i^—Co1—O1^i^	92.79 (7)	N11—C11—H11	118.5
O1—Co1—O1^i^	180.0	C12—C11—H11	118.5
N1—Co1—N11^i^	90.64 (7)	C13—C12—C11	118.36 (19)
N1^i^—Co1—N11^i^	89.36 (7)	C13—C12—H12	120.8
O1—Co1—N11^i^	89.26 (6)	C11—C12—H12	120.8
O1^i^—Co1—N11^i^	90.74 (6)	C12—C13—C14	119.73 (19)
N1—Co1—N11	89.36 (7)	C12—C13—C16	120.8 (2)
N1^i^—Co1—N11	90.64 (7)	C14—C13—C16	119.4 (2)
O1—Co1—N11	90.74 (6)	C15—C14—C13	118.0 (2)
O1^i^—Co1—N11	89.26 (6)	C15—C14—H14	121.0
N11^i^—Co1—N11	180.0	C13—C14—H14	121.0
C1—N1—Co1	166.48 (18)	N11—C15—C14	123.42 (18)
N1—C1—S1	179.7 (2)	N11—C15—H15	118.3
Co1—O1—H1A	124 (2)	C14—C15—H15	118.3
Co1—O1—H1B	127 (2)	N12—C16—C13	178.7 (5)
H1A—O1—H1B	107 (3)		

Symmetry code: (i) −*x*+1, −*y*+1, −*z*.

### Diaquadithiocyanatobis(pyridine-4-carbonitrile)cobalt(II) Hydrogen-bond geometry (Å, º)

**Table:**

*D*—H···*A*	*D*—H	H···*A*	*D*···*A*	*D*—H···*A*
O1—H1*A*···S1^ii^	0.82 (2)	2.50 (2)	3.2272 (17)	150 (3)
O1—H1*B*···S1^iii^	0.81 (2)	2.53 (2)	3.3227 (16)	168 (3)
C15—H15···N12^iv^	0.93	2.45	3.144 (3)	132

Symmetry codes: (ii) −*x*+1, −*y*+1, −*z*+1; (iii) −*x*+1, *y*−1/2, −*z*+1/2; (iv) −*x*+2, *y*−1/2, −*z*+1/2.
